# PuraStat as an Adjunct in the Management of Acute Gastric Variceal Haemorrhage: A Case Report

**DOI:** 10.7759/cureus.117998

**Published:** 2026-10-15

**Authors:** Abhishek Mappilassery Ouseph, Divine Stephen, Ammar Al-Rifaie

**Affiliations:** 1 Gastroenterology, Chesterfield Royal Hospital, Chesterfield, GBR; 2 General Internal Medicine, Chesterfield Royal Hospital, Chesterfield, GBR

**Keywords:** decompensated cirrhosis, endoscopic band ligation, endoscopic haemostasis, gastric varices, haemostatic gel, liver cirrhosis, portal hypertension, purastat, upper gi bleeding, variceal haemorrhage

## Abstract

Acute variceal haemorrhage is a life-threatening complication of portal hypertension. Standard management includes haemodynamic resuscitation, vasoactive therapy, antibiotic prophylaxis, and urgent endoscopic haemostasis. We report a case in which PuraStat, a self-assembling peptide haemostatic gel, was used as an adjunct during active gastric variceal bleeding to provide temporary haemostatic control and improve endoscopic conditions for subsequent intervention. Following PuraStat application, haemostasis was achieved. This case supports a potential adjunctive role for PuraStat in selected patients with acute gastric variceal haemorrhage when immediate control proves difficult. PuraStat's temporary haemostatic effect may provide a bridge until more definitive management can be arranged in the correct setting.

## Introduction

Portal hypertension, most commonly caused by cirrhosis, leads to the formation of portosystemic collaterals, including gastric and oesophageal varices. Gastric varices are less common than oesophageal varices but, when they bleed, the haemorrhage is often more severe and harder to control, with higher rebleeding and mortality rates [1]. Isolated gastric varices of the fundus (IGV1 in the Sarin classification) are of particular concern. Patients typically present with haematemesis and/or melaena, often with haemodynamic compromise, in the setting of known or previously undiagnosed chronic liver disease [1].

Acute variceal haemorrhage is a medical emergency associated with significant morbidity and mortality in patients with portal hypertension. Management typically includes haemodynamic resuscitation, vasoactive therapy, antibiotic prophylaxis, and urgent endoscopic intervention, most commonly variceal band ligation for oesophageal varices and gluing for gastric varices [1-4]. In cases of brisk or difficult-to-control bleeding, adjunctive haemostatic agents may be considered to aid temporary control and improve endoscopic visualisation. Balloon tamponade with a Sengstaken-Blakemore tube has been used as a temporary bridge to definitive therapy in refractory variceal bleeding, although it is of limited benefit in isolated gastric varices, and few options exist for temporary endoscopic control of bleeding in this setting [5].

PuraStat is a self-assembling peptide haemostatic gel designed to form a mechanical barrier over bleeding surfaces. Its use in gastrointestinal bleeding has been described in a number of settings, including peptic ulcer disease and post-polypectomy haemorrhage [6-9]. However, its role in acute variceal haemorrhage is less well established, although a case report of its use in oesophageal variceal bleeding suggested added benefit in controlling haemorrhage [10]. We describe a case in which PuraStat was used as an adjunct to control bleeding temporarily in acute gastric variceal bleeding, with subsequent haemostasis achieved.

## Case presentation

A 62-year-old male with a background history of decompensated alcohol-related liver disease presented to the Emergency Department with haematemesis and melaena. On arrival, the patient was haemodynamically stable, with a blood pressure of 141/53 mmHg, heart rate of 91 bpm, and an initial haemoglobin of 71 g/L (local laboratory reference range: 130-170 g/L). The Glasgow-Blatchford score was 13 [11]. 

Resuscitation was commenced with intravenous access and blood transfusion. Vasoactive therapy with terlipressin was initiated [12]. Prophylactic intravenous ceftriaxone was commenced in line with standard practice for acute variceal haemorrhage [1,2].

Upper gastrointestinal endoscopy was performed on day 2 of admission, approximately 31 hours after presentation, with patient consent. This was later than the recommended 12 hours, as the gastroenterology team was not contacted at the time of the patient's initial assessment in the Emergency Department. Endoscopic assessment identified a bleeding isolated gastric varix type 1 (IGV1, Sarin classification) arising from a central punctum in the gastric fundus (Figures 1, 2). No other bleeding source was identified.

**Figure 1:**
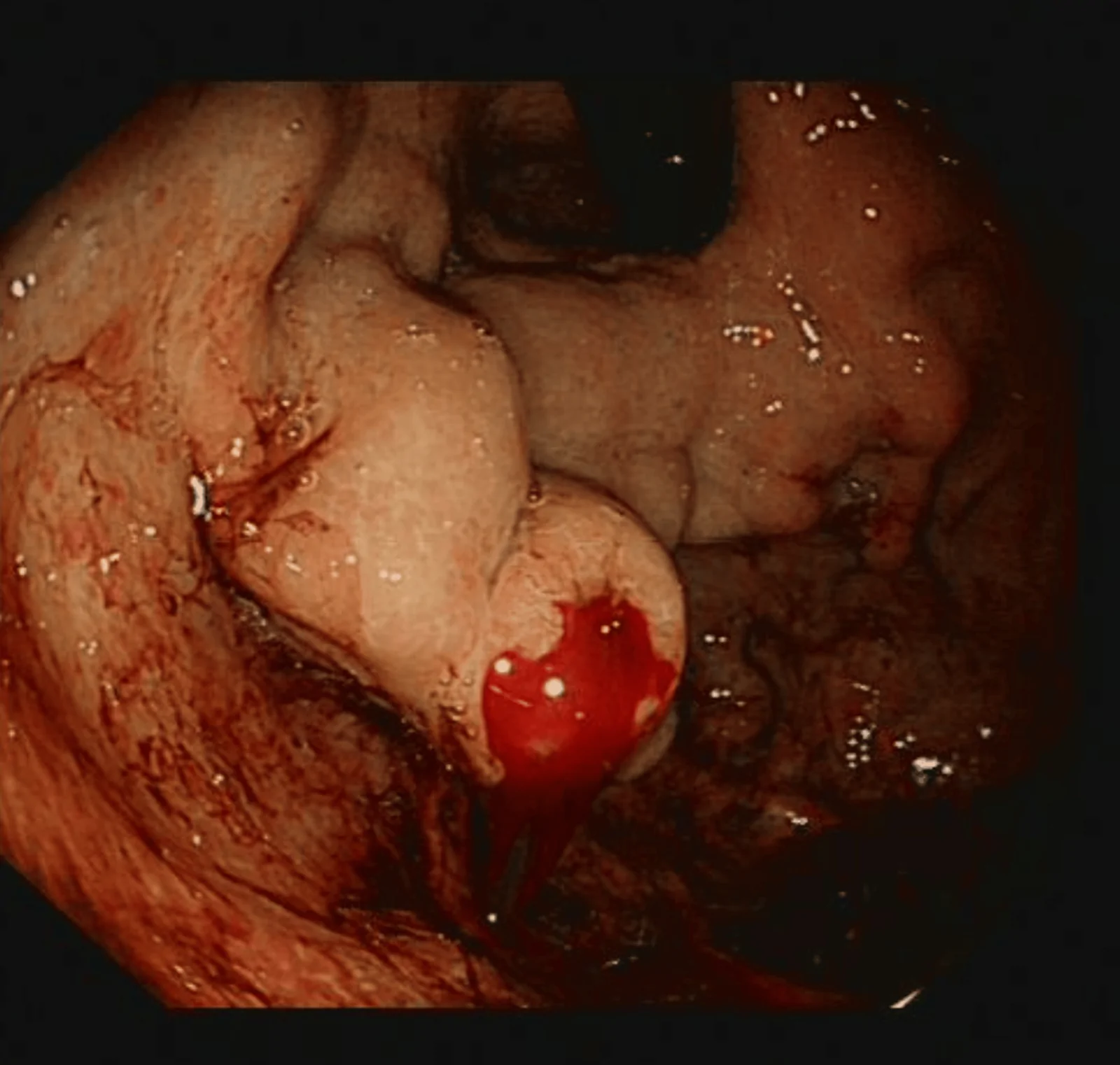
Endoscopic view of the bleeding gastric varix at index gastroscopy, prior to PuraStat application.

**Figure 2:**
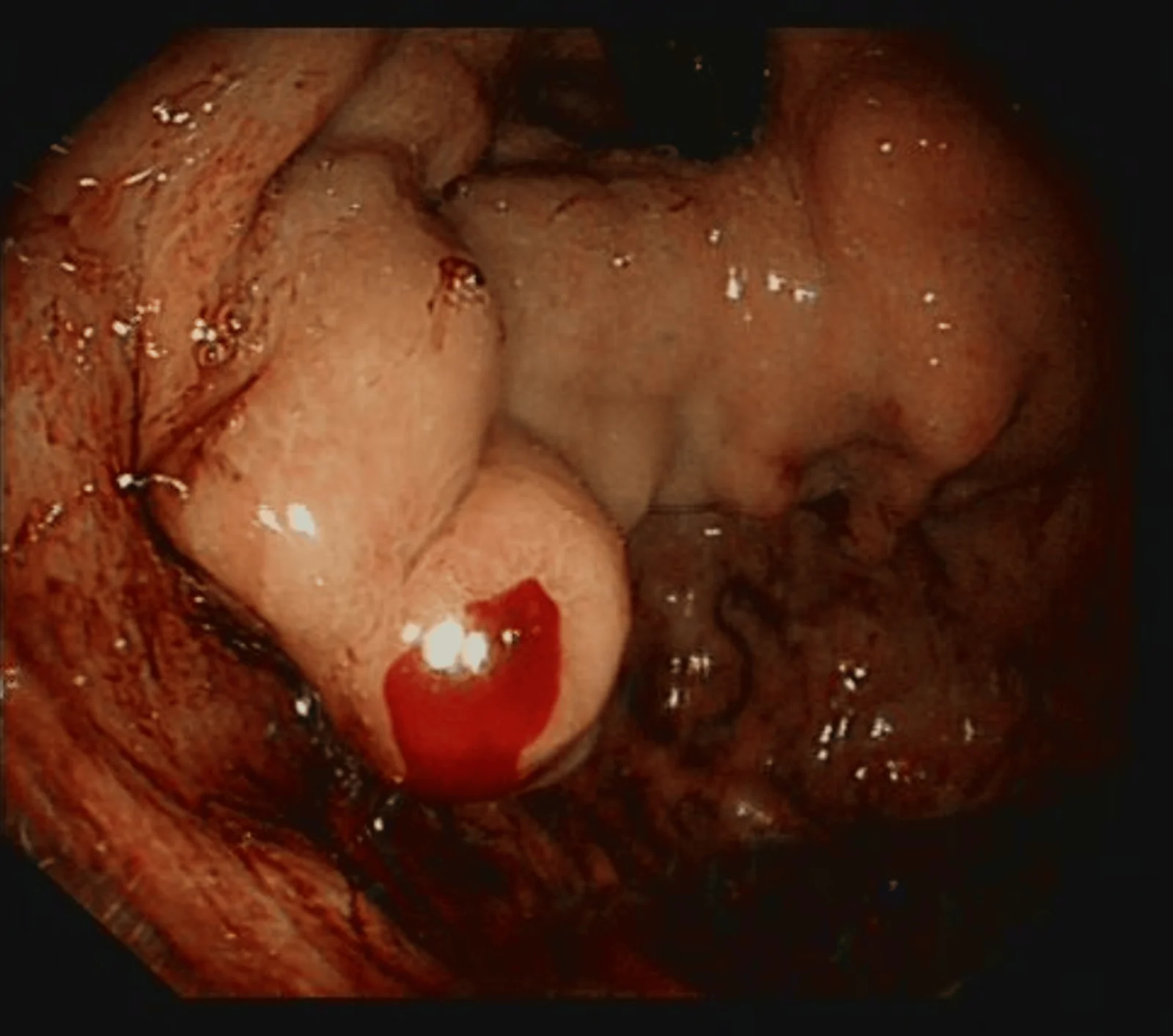
Bleeding gastric varix with active haemorrhage from the central punctum, prior to PuraStat application.

Although the patient remained haemodynamically stable, he was agitated and inadequately sedated despite 3 mg of intravenous midazolam. This precluded safe endoscopic cyanoacrylate injection, which requires precision and a settled patient. As the procedure took place out of hours, a decision was made to defer glue injection to a subsequent gastroscopy under general anaesthesia in theatres. As a temporising measure, 3 ml of PuraStat haemostatic gel was applied endoscopically to the bleeding varix. This achieved satisfactory local haemostasis, with cessation of active bleeding confirmed before the end of the procedure (Figure 3). Haemostasis was maintained for approximately three hours until the second gastroscopy.

**Figure 3:**
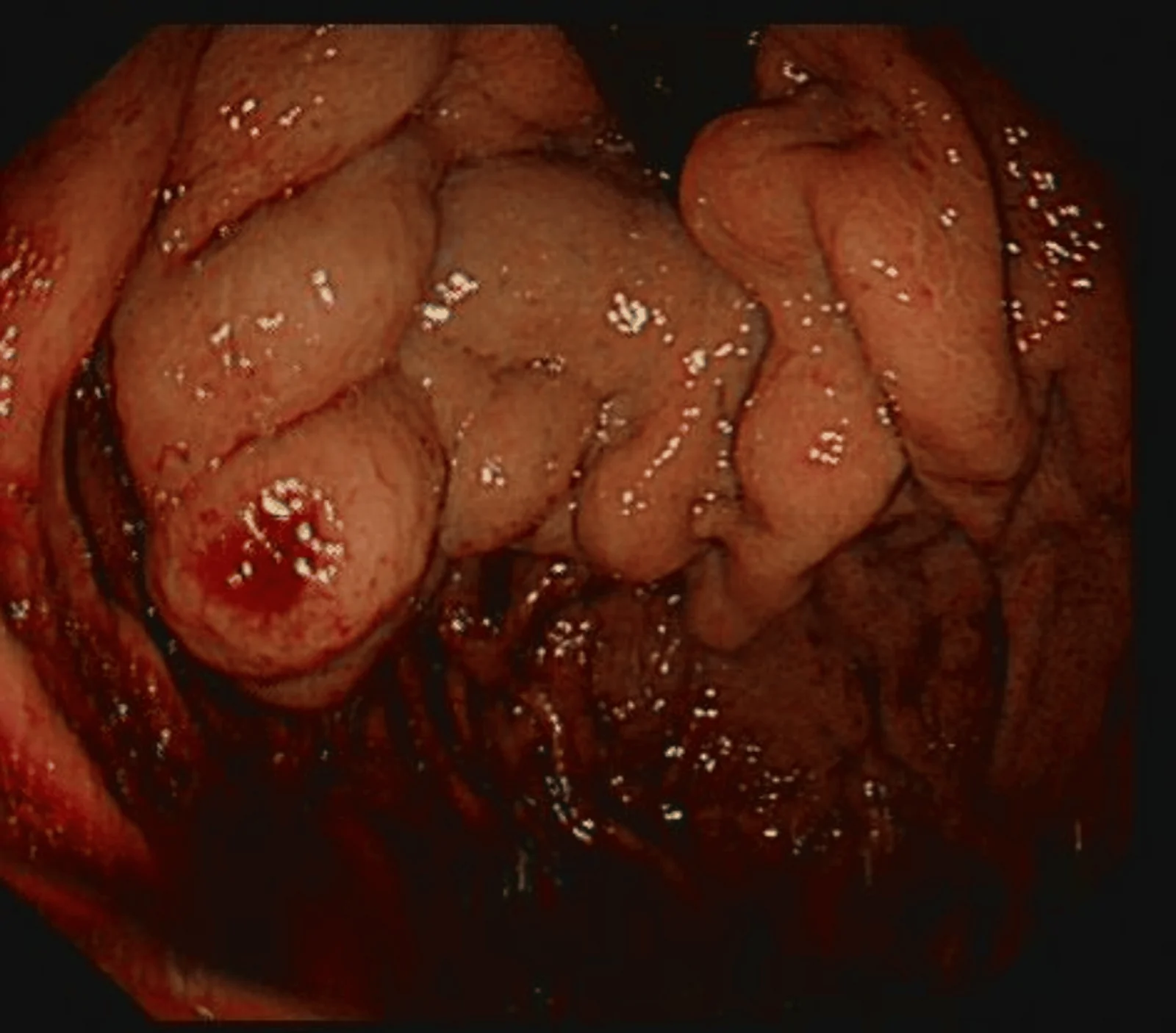
Endoscopic appearance of the gastric varix following PuraStat application, with reduction in active bleeding.

A second gastroscopy was subsequently performed under general anaesthesia. Endoscopic assessment at this stage demonstrated the gastric varix with stigmata of recent haemorrhage but no active bleeding. Cyanoacrylate glue (0.5 ml) was injected adjacent to the variceal punctum per local protocol [3,4]. The injection catheter was then used to apply direct tamponade pressure to the injection site. Following approximately two minutes of sustained pressure, haemostasis was achieved and confirmed. The varix subsequently blanched, consistent with successful obliteration.

Following the procedure, the patient was monitored in the ward and remained haemodynamically stable. No immediate adverse events related to the use of PuraStat were observed. The patient was discharged on day 10 with follow-up in the Gastroenterology Clinic.

## Discussion

This single case illustrates a possible adjunctive role for PuraStat in acute gastric variceal bleeding when adequate preparation and/or sedation alone are not sufficient to permit safe endoscopic glue injection. In our case, PuraStat appeared to provide temporary haemostatic support, helping to reduce active bleeding and improve procedural conditions until definitive haemostasis was achieved.

The key proposed advantage of PuraStat in this setting is its ability to form a transparent, biocompatible physical barrier over the bleeding site via self-assembling peptide technology. This may be particularly useful when bleeding is diffuse or endoscopic visibility is significantly impaired. As PuraStat is applied topically rather than injected into the vessel, the risk of embolisation associated with cyanoacrylate injection is theoretically lower, and it has a favourable safety profile in the non-variceal gastrointestinal haemorrhage literature [8,9,13]. Its safety in variceal bleeding has not been established.

It should be emphasised, however, that PuraStat is not intended to replace guideline-based management of variceal haemorrhage, which mandates vasoactive therapy, antibiotic prophylaxis, and definitive endoscopic treatment [1,2]. Its role, as illustrated here, is as a temporising adjunct rather than a standalone therapy. Notably, PuraStat degrades naturally and would not be expected to provide durable haemostasis in the context of ongoing portal hypertension without definitive treatment of the variceal source [1].

Published experience with PuraStat in variceal haemorrhage remains limited. A small number of case reports and series have suggested a potential benefit, but robust prospective data are lacking [8-10]. This single case adds to the limited existing literature and suggests that PuraStat may have a niche role in selected patients with acute variceal bleeding, particularly where immediate control is difficult. Further studies are required to establish efficacy, safety, and appropriate patient selection criteria in this context.

This report has several limitations. It describes a single patient, so no conclusions can be drawn about efficacy or safety. PuraStat is best established for oozing and small-vessel bleeding, and its reliability in high-flow gastric varices is unproven. The duration of its haemostatic effect is unknown, as cyanoacrylate was injected within hours. Haemostasis was maintained for approximately three hours in this case, but this cannot be generalised. The safe and effective volume in variceal bleeding is not defined; 3 ml was used here without adverse events. Finally, endoscopy was performed approximately 31 hours after presentation, later than the recommended 12 hours. Earlier endoscopy and early involvement of the gastroenterology team are the standard of care, and PuraStat should not be seen as a substitute for these.

## Conclusions

In this single case, PuraStat appeared to be a useful temporising adjunct in acute gastric variceal haemorrhage to provide temporary bleeding control and give more time to facilitate definitive haemostasis. This case adds to the limited existing literature on its use in complex upper gastrointestinal haemorrhage. Prospective studies with larger patient cohorts are needed to define its place within the management algorithm for acute variceal bleeding.
